# Temperature and Frequency Dependence of Human Cerebrospinal Fluid Dielectric Parameters

**DOI:** 10.3390/s24227394

**Published:** 2024-11-20

**Authors:** Weice Wang, Mingxu Zhu, Benyuan Liu, Weichen Li, Yu Wang, Junyao Li, Qingdong Guo, Fang Du, Canhua Xu, Xuetao Shi

**Affiliations:** 1 Department of Biomedical Engineering, Shaanxi Provincial Key Laboratory of Bioelectromagnetic Detection and Intelligent Perception, Air Force Medical University, Xi’an 710032, China; wangweice@fmmu.edu.cn (W.W.); mingxuzhu@fmmu.edu.cn (M.Z.); junyaoli@fmmu.edu.cn (J.L.); 2 Department of Radiology, Functional and Molecular Imaging Key Lab of Shaanxi Province, Tangdu Hospital, Air Force Medical University, Xi’an 710000, China; oliver2015@fmmu.edu.cn; 3 Institute of Medical Research, Northwestern Polytechnical University, Xi’an 710072, China; wyu_nwpu@mail.nwpu.edu.cn; 4 Department of Neurosurgery, Xijing Hospital, Air Force Medical University, Xi’an 710032, China; 5 Department of Neurology, Xijing Hospital, Air Force Medical University, Xi’an 710032, China

**Keywords:** cerebrospinal fluid, frequency and temperature dependence, electrical impedance spectroscopy, dielectric properties, bioelectromagnetism

## Abstract

Accurate human cerebrospinal fluid (CSF) dielectric parameters are critical for biological electromagnetic applications such as the electromagnetic field modelling of the human brain, the localization and intensity assessment of electrical generators in the brain, and electromagnetic protection. To detect brain damage signals during temperature changes by electrical impedance tomography (EIT), the change in CSF dielectric parameters with frequency (10 Hz–100 MHz) and temperature (17–39 °C) was investigated. A Debye model was first established to capture the complex impedance frequency and temperature characteristics. Furthermore, the receiver operating characteristic (ROC) analysis based on the dielectric parameters of normal and diseased CSF was carried out to identify lesions. The Debye model’s characteristic *f*_c_ parameters linearly increased with increasing temperature (*R*^2^ = 0.989), and *R*_0_ and *R*_1_ linearly decreased (*R*^2^ = 0.990). The final established formula can calculate the complex impedivity of CSF with a maximum fitting error of 3.79%. Furthermore, the ROC based on the real part of impedivity at 10 Hz and 17 °C yielded an area under the curve (AUC) of 0.898 with a specificity of 0.889 and a sensitivity of 0.944. These findings are expected to facilitate the application of electromagnetic technology, such as disease diagnosis, specific absorption rate calculation, and biosensor design.

## 1. Introduction

The dielectric parameters of biological tissues are subject to significant influence from a number of factors, including pathological state, temperature, frequency, and tissue type. The electrical parameters of the brain are of diagnostic value for many physiological and pathological conditions [1,2,3,4]. Brain damage resulting from ischemia and hypoxia represents a severe and prevalent complication of cardiopulmonary bypass (CPB) procedures [5,6]. The potential of EIT to monitor and alert early to the risk of brain damage during deep hypothermic circulatory arrest in CPB was firstly demonstrated by our team [7]. However, there is also a risk of brain damage during the temperature change process in CPB. To further improve the monitoring effectiveness, it is essential to extract the brain injury-related electrical signal during temperature change, which is significantly influenced by temperature. To address this issue, it is crucial to understand the temperature dependence of the dielectric parameters of brain tissue.

The extant literature on the broadband frequency and temperature dependence of the electrical properties of brain tissues is insufficient. In order to establish a more accurate head model, Latikka et al. and Koessler et al. measured the in vivo conductivity of human grey matter, white matter, CSF, brain tumours, and epileptogenic zones at 50 kHz [8,9]. This provided valuable data on the dielectric parameters of living brain tissue, which constitutes a significant contribution to the field. The Gabriel team conducted a comprehensive and systematic investigation into the dielectric properties of various brain components, including CSF, grey matter, white matter, the spinal cord, and the dura mater [10,11,12,13,14]. The widely used broadband dielectric parameters of brain tissue are based on the seminal work of Gabriel et al. However, it should be noted that the impact of temperature on the parameters was not considered. To address the issue of electric field distortion and location errors resulting from inaccurate CSF dielectric parameters, Stephen et al. conducted a study examining the variation in CSF conductivity with frequency (10 Hz–10 kHz) at 25 °C and 37 °C [15]. This study was the first to discover and quantify the temperature characteristics of CSF dielectric parameters. However, the temperature points examined by the authors were insufficient for the purposes of this study. In order to utilize brain impedance for monitoring of the recovery process after hypothermia treatment in neonates with hypoxia, Barbara et al. conducted the inaugural in vivo study about the overall impedance of piglet brains at 32–39 °C and 4–1012 kHz [16]. However, the authors did not consider the shielding effect of the skull on the current, which meant the study may not have accurately reflected the change rule of the intracranial tissues’ electrical parameters with temperature and frequency. Consequently, it is essential to investigate the temperature and frequency dependence of each brain tissue’s electrical parameters individually.

Firstly, it is notable that there is currently a paucity of available literature on the temperature-dependent behaviour of the CSF dielectric parameters. Secondly, the CSF constitutes approximately 10% of the brain’s volume [17]. The compensatory mechanisms of CSF result in its redistribution when brain diseases occur and intracranial pressure rises. This alteration exerts a complex effect on the overall dielectric parameters of the brain [18]. Finally, studies have demonstrated that the electromagnetic field of the brain is highly sensitive to alterations in the dielectric parameters of CSF. Inaccurate CSF dielectric parameters can result in distortion of the electromagnetic field of the head and errors in the location and intensity evaluation of electrical generators in the brain [15,19,20,21]. Therefore, there is an urgent need to master the frequency and temperature characteristics of CSF electrical parameters.

This study was conducted in the context of the significant impact of temperature changes on the brain’s dielectric parameters during CPB. To begin with, the dielectric parameters of human CSF were measured over a frequency range of 10 Hz to 100 MHz at temperatures between 17 and 39 degrees Celsius, in accordance with the methodology previously established by our research group [22]. Then, the influence of cooling and reheating processes on CSF complex impedivity was examined. Subsequently, a functional model was constructed to describe the relationship among the dielectric parameters of human CSF, temperature, and frequency. Finally, the ability of the impedance method to identify CSF lesions was also explored. This study establishes a foundation for accurate modelling of the frequency–temperature relevance of overall brain electrical parameters, facilitating the future detection and early identification of brain injuries during CPB using EIT.

## 2. Materials and Methods

### 2.1. Sample Collection

The subjects were patients requiring lumbar puncture for CSF analysis at the Department of Neurology at Xijing Hospital. The patients were randomly assigned to the study groups with an average age of 59.6 ± 6.3 years, and the gender distribution was balanced. The CSF samples were divided into two parts, one for routine biochemical examination to differentiate between normal and pathological CSF. The other part was used for impedance measurement and was further divided into groups. Group A: 7 normal CSF samples were studied to determine the impact of cooling and reheating on their electrical parameters. Group B: The complex impedivity of 18 normal CSF samples was investigated for temperature (17–39 °C) and frequency (10 Hz–100 MHz) dependence. Group C consisted of 18 pathological CSF samples which were used to investigate the differences between normal and pathological CSF dielectric parameters at different temperatures and frequencies.

### 2.2. Impedance Measurement

Based on the measurement platform developed by our group [22], the dielectric parameters of CSF were measured at 10 Hz–100 MHz. The frequency sweeps were performed in logarithmic form, with 10 data points collected at every 10th frequency, resulting in a total of 71 frequency points. The temperature of the samples was controlled using a water bath with target temperatures of 17 °C, 22 °C, 27 °C, 32 °C, 37 °C, and 39 °C.

The impedance analyzer measured the real (*Re*) and imaginary (*Im*) data of the complex impedance. The impedivity real (*ρ*_real_) and imaginary (*ρ*_imaginary_) parts of CSF can be calculated from the geometry of the impedance measuring box [22] (as shown in Equations (1) and (2)), where *A* is the cross-sectional area of the CSF, and *d* is the length of the CSF.

(1) $$\rho_{real}=Re\times \frac{A}{d}\times 100(\Omega \cdot cm)$$

(2) $$\rho_{imaginary}=Im\times \frac{A}{d}\times 100(\Omega \cdot cm)$$

### 2.3. Functional Model of the Frequency–Temperature-Relevant CSF Dielectric Parameters

**Figure 1:**
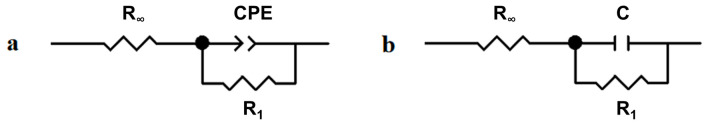
(**a**) Circuit with a constant phase angle unit and a resistor in parallel configuration; (**b**) circuit with a resistor and a capacitance in parallel configuration.

In order to quantify the functional relationships among the CSF dielectric parameters, frequency, and temperature, the first step was to construct an equivalent circuit model to describe the frequency dependence of the complex impedance of human CSF at 10 Hz–100 MHz. Therefore, a Cole–Cole equivalent circuit model was constructed, containing of a constant phase angle unit and a resistor in parallel configuration (Figure 1a). Additionally, a “three-element” equivalent circuit model with a resistor and a capacitance in parallel configuration was constructed (Figure 1b). The model parameters were derived by ZsimpWin software. And Equation (3) was used to assess the relative error between the calculated and the measured dielectric parameters of the CSF (*Z* indicates complex impedance or complex impedivity).

(3) $$Err=\frac{\left|Z_{measured\;}|-|Z_{fit}\right|}{\left|Z_{measured}\right|}*100\%$$

The optimal circuit model was identified according to Equation (3). Subsequently, an analysis of the temperature dependence of each model parameter was then conducted. This analysis aimed to formulate a comprehensive mathematical function capable of expressing the relationship among frequency, CSF’s complex impedance, and temperature. Finally, the complex impedivity of the tissue under investigation was calculated from the known geometric information.

### 2.4. An Exploratory Study of Bioimpedance Techniques for the Identification of CSF Lesions

To explore whether the impedance method can be used to identify CSF lesions, this study compared the dielectric parameters of normal and pathological CSF at different temperatures and frequencies. The Shapiro–Wilk normality test was first applied to the real and imaginary parts of impedivity within the frequency range of 10 Hz–100 MHz and the temperature range of 17–39 °C at a significance level of *α* = 0.05. For the complex impedivity that exhibited a normal distribution, independent-sample t-tests were conducted. In the case of the complex impedivity that did not conform to a normal distribution, Mann–Whitney U-tests were employed. This enabled the identification of the frequency and temperature points at which the complex impedivity of the two types of CSF exhibited significant differences. In order to evaluate the effectiveness of accurately differentiating normal and pathological CSF, this paper plotted the ROC curves using the dielectric data with significant differences as the independent variable and the type of CSF as the dependent variable (normal = 0, pathological = 1). Furthermore, the AUC, sensitivity, and specificity were also calculated.

## 3. Results

### 3.1. Influence of Cooling and Reheating Processes on CSF’s Dielectric Characteristics

**Figure 2:**
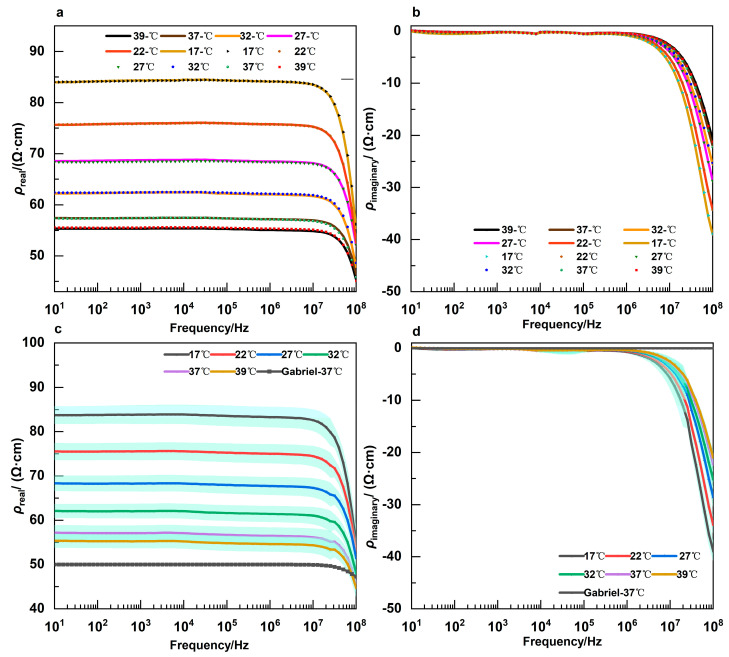
(**a**) Changes in the average real parts of impedivity across different frequencies throughout the cooling and reheating progress; (**b**) changes in the average imaginary parts of impedivity across different frequencies throughout the cooling and reheating progress; the temperatures of 39- °C, 37- °C, 32- °C, 27- °C, 22- °C, and 17- °C correspond to the cooling progress and the temperatures of 17 °C, 22 °C, 27 °C, 32 °C, 37 °C, and 39 °C correspond to the reheating progress. (**c**) Trend of the average real parts of impedivity versus frequency at different temperature levels. (error bars represent S.D.); (**d**) trend of the average imaginary parts of impedivity versus frequency at different temperature levels. (Error bars represent S.D.).

CPB usually entails cooling and reheating processes. Nevertheless, the consistency of CSF dielectric parameters has not received sufficient scrutiny at the same temperature during cooling and reheating. Accordingly, an investigation was conducted into the real and imaginary parts of the normal CSF impedivity across a frequency range of 10 Hz to 100 MHz during cooling and reheating. Figure 2a,b illustrate the changes in the average values of the imaginary and real parts as a function of frequency at each temperature throughout the initial cooling and subsequent reheating phases, respectively. The Shapiro–Wilk normality test was conducted on the real and imaginary parts at each frequency and temperature during cooling and reheating. Paired t-tests were performed at the corresponding temperatures and frequencies for data that conformed to a normal distribution, whereas the non-parametric Wilcoxon-signed rank test was employed for those that did not conform to the normal distribution. The results demonstrated that the *p* > 0.08, indicating that there was no statistically significant difference in the dielectric parameters during cooling and reheating.

### 3.2. Dielectric Parameters of CSF as a Function of Temperature

The aforementioned study demonstrated that the cooling and reheating cycles exerted no significant impact on the CSF dielectric characteristics. To avoid the possibility of tissue inactivation resulting from prolonged ex vivo exposure, the experimental process was simplified. The study analyzed the electrical parameters that changed with frequency throughout the heating process using a sample set of 18 normal CSF samples, with the total time of 43.9 ± 4.2 min.

Figure 2c,d demonstrate that the real and imaginary parts of normal CSF undergo frequency-dependent alterations at different temperatures. Moreover, the changes at 37 °C, as reported by Gabriel et al. [10,11,12], are also depicted. Notwithstanding the disparate values of the impedivity real and imaginary parts at various temperatures, their frequency-dependent behaviour remained consistent. The real parts exhibited minimal variation at lower frequencies. A notable reduction in the real parts was observed after 10 MHz. Furthermore, it was observed that with rising temperatures, the frequency point at which the steep decline of the real parts commenced shifted progressively to higher values. With regard to the imaginary parts, values were nearly zero within the range of 10 Hz to 1 MHz. Beyond this range, as frequency increased, a downward trend was observed in the imaginary parts. Moreover, the imaginary parts increased at the same frequency point as the temperature increased.

### 3.3. A Model Describing the Broadband Frequency–Temperature-Dependent Dielectric Parameters of CSF

This study presented two equivalent circuits, depicted in Figure 1, which were designed to characterize the frequency-dependent behaviour of the complex impedance of CSF across 10 Hz to 100 MHz. The parameter *R*_∞_ < 0, extracted from Figure 1a, did not align with the expected practical conditions. Moreover, the relative error of the circuit depicted in Figure 1b was found to be 1.26% when fitting the complex impedance of CSF at 37 °C. Therefore, the equivalent circuit presented in Figure 1b was chosen to represent the frequency response of complex impedance at different temperatures.

To obtain the characteristic parameters indicative of physiological and pathological conditions, a first-order Debye model [23] was formulated to depict the frequency-dependent behaviour of the CSF’s complex impedance across different temperatures, as depicted in Equations (4) and (5).

(4) $$Impedance(f,T)=R_{\infty }(T)+\frac{R_{1}(T)}{1+j\frac{f}{f_{c}(T)}}$$

(5) $$f_{c}=\frac{1}{2*\mathrm{PI}\times R_{1}(T)\times C(T)}$$

In this model, the variable *f* represents the frequency within the range of 10 Hz–100 MHz, while *T* denotes the temperature within the range of 17–39 °C. The term *R*_∞_(*T*) denotes the impedance at the high frequency, and *R*_1_(*T*) represents the impedance variation within the dispersion region. Finally, *f*_c_(*T*) denotes the characteristic frequency in the dispersion region. By the above parameters, we could extract *R*_0_(*T*) = *R*_∞_(*T*) + *R*_1_(*T*), which was found to be closely correlated with the composition of substances within the extracellular fluid and represents the impedance under DC.

**Figure 3:**
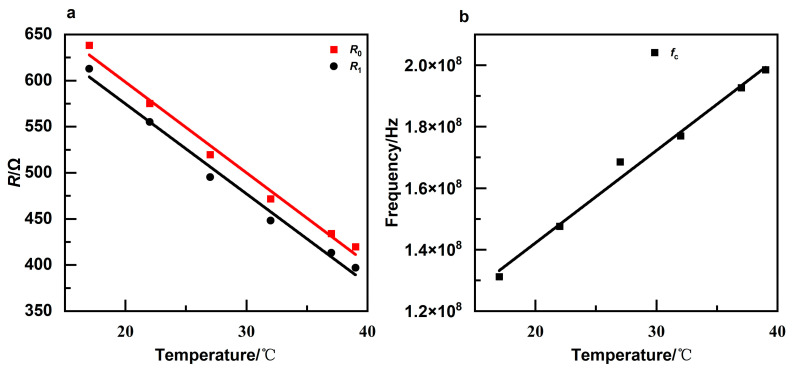
(**a**) Plot of characteristic parameters *R*_0_, *R*_1_ versus temperature, with data points and corresponding linear fitting lines; (**b**) plot of characteristic frequencies *f*_c_ versus temperature, with data points and linear fitting line.

As illustrated in Figure 3a,b, curve fitting was performed to analyze the correlation between each temperature and the characteristic parameter, demonstrating a robust linear relationship. The linear fitting coefficients for each parameter as a function of temperature, along with the coefficient of determination, are presented in Table 1.

With the correlation between the characteristic parameters and temperature determined, the Debye model was established to describe the relationships among complex impedance, temperature, and frequency. Based on the geometry of the tissue, the final expression for the complex impedivity was derived and is presented in Equation (6). In the equation, the variables are defined as follows: *ρ* represents complex impedivity, *T* represents any temperature within the range of 17 to 39 °C, and *f* represents any frequency in the range of 10 Hz to 100 MHz.

(6) $$\rho (f,T)=-0.010\times T+\frac{-1.277\times T+100.793}{1+j\frac{f}{3007513.102\times T+82067882.929}}+3.295(\Omega \cdot \mathrm{cm})$$

**Figure 4:**
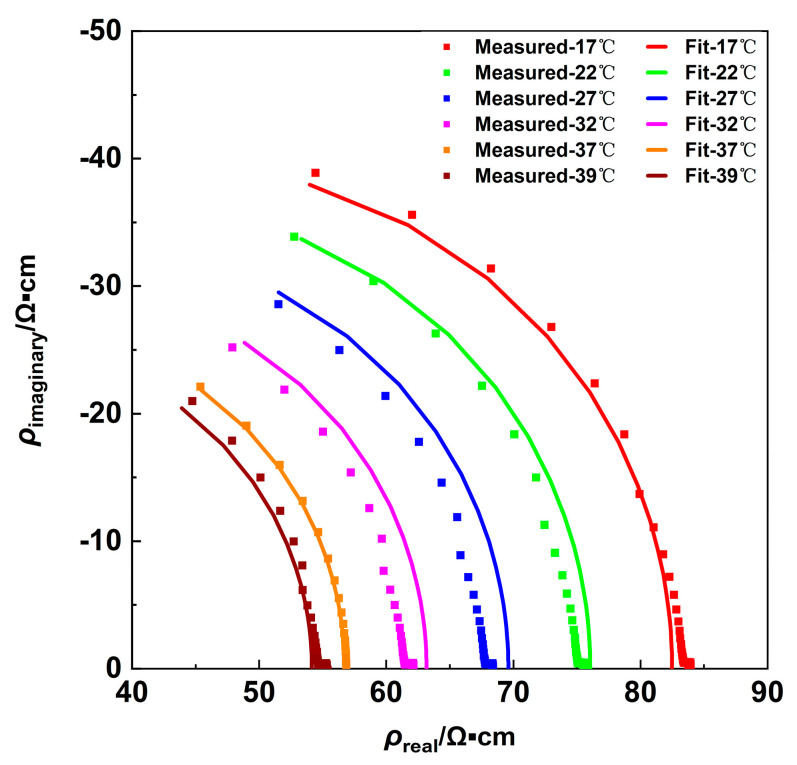
Measured impedivity data (points) and corresponding model fitting (lines) at various temperatures.

As illustrated in Figure 4, a Nyquist plot [24] with the real part as the horizontal coordinate and the imaginary part as the vertical coordinate was plotted. In the plot, the data points represent the measured complex impedivity at different temperatures and frequencies, while the solid lines indicate the values obtained from the fit using Equation (6). The maximum relative error was 3.79%, thereby demonstrating a strong concordance between the measured and the fitted data.

### 3.4. Difference in the Dielectric Parameters of Normal and Pathological CSF

**Figure 5:**
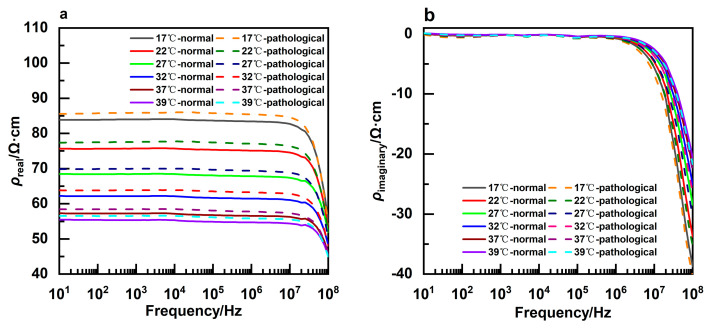
Variation in the impedivity real parts (**a**) and imaginary parts (**b**) of normal and pathological CSF with frequency at different temperatures.

Eighteen pathological CSF samples were screened by routine CSF examination and biochemical examination. The dielectric parameters of normal and pathological CSF were compared at different temperatures and frequencies (shown in Figure 5). It was indicated that the average values of the real part of impedivity of pathological CSF were greater than those of normal CSF at the same temperature. Furthermore, the difference between the two types of CSF became smaller when the frequency exceeded 10 MHz. The mean impedivity imaginary part of pathological CSF was found to be less than that of normal CSF at the same temperature. The dielectric parameter with the most significant difference was the real part of impedivity at 10 Hz and 17 °C, with a *p*-value of 0.005.

**Figure 6:**
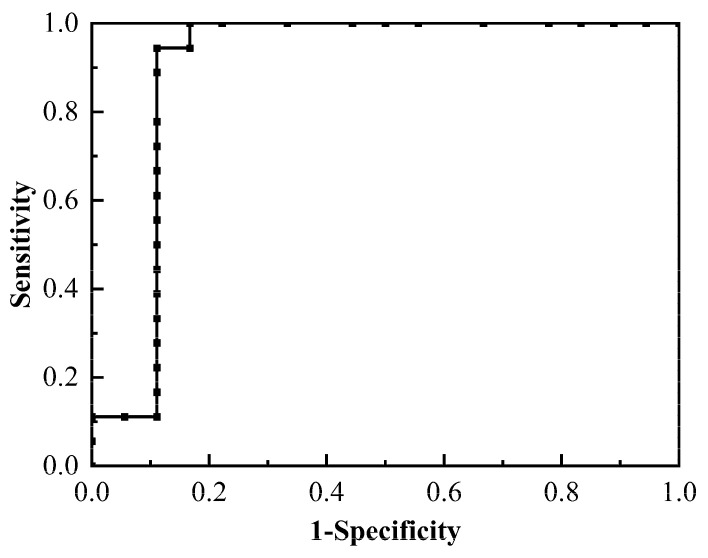
ROC curve of real part of impedivity at 10 Hz, 17 °C for identification of CSF pathological states.

The ROC curve shown in Figure 6 was generated using the real part of impedivity at 10 Hz and 17 °C as the independent variable. The AUC was 0.898, and the sensitivity was 0.944, while the specificity was 0.889 when the threshold value of 84.515 was employed to discriminate between healthy and diseased CSF.

## 4. Discussion

When using EIT to monitor and warn against brain damage during CPB, our research group found that temperature change has a significant impact on the extraction of impedance signals related to brain damage [7]. In order to extract the signals of interest, it is first necessary to understand the temperature and frequency characteristics of the dielectric parameters of key brain tissues. Many studies have shown that CSF, as a key component of the brain, plays a significant role in determining the overall dielectric characteristics of the brain [15,17,18,19,20,21]. However, there is a paucity of studies investigating the temperature and frequency dependence of CSF dielectric properties. Consequently, further investigation into the temperature- and frequency-dependent characteristics of CSF dielectric parameters is warranted.

Physiological and pathological changes can be reflected in the dielectric parameters within 10 Hz–100 MHz, and many electromagnetic technologies also operate in this frequency range [25]. It is therefore of great scientific significance and application value to investigate the dielectric parameters at 10 Hz–100 MHz. During the cooling phase of the CPB, the patient’s temperature can reach as low as 17 °C [26]. In the postoperative period, the patient may present with a fever. Therefore, the temperature range of the study was set at 17–39 °C. It was unclear whether the cooling and reheating process would cause irreversible alterations to the dielectric properties of CSF, which could potentially have led to undesirable interference in the study of the temperature dependence. Accordingly, this study compared the real and imaginary parts of impedivity during the cooling and reheating process at the same temperature and frequency. The results showed that the impact of this process upon the dielectric parameters of CSF is reversible. It is hypothesized that the primary components of the CSF are ions, and the temperature primarily affects the extent of ions’ ionization. The process of ionization is reversible within this temperature range. Concurrently, cells, proteins, and other active substances are unlikely to be inactivated and denatured within this temperature range. This finding suggests that the experimental procedure can be further simplified, whereby only variations in the dielectric parameters of CSF during unidirectional temperature changes are studied. In this way, the effect of inactivation in vitro can be reduced and the temperature-dependent nature of the dielectric parameters of in vivo CSF can be accurately reflected.

At all measured temperatures, CSF exhibited similar frequency-dependent behaviour in both the impedivity real and imaginary components. In the low-frequency range (10 Hz–10 MHz), the real parts remained unchanged, and the imaginary parts approached 0. This phenomenon may be attributed to the fact that normal CSF is primarily composed of water (99%), sodium ions (155 mM/L), chloride ions (125 mM/L), and bicarbonate (28 mM/L) [17], and therefore, CSF shows good ’pure resistor’ dielectric properties in the low-frequency range. As the frequency increases, the current interacts with the trace cells, proteins, sugars, etc., to produce dispersion [27], resulting in a decrease in the real and imaginary parts. It can thus be concluded that the principal mechanism by which temperature affects dielectric parameters at lower frequencies is to increase the degree of ionization, which leads to an increase in the concentration of ions in the CSF. As the frequency increases, the effect of temperature on the dielectric parameters can be divided into two main categories. Firstly, temperature alters the degree of ionization, and secondly, it affects the activity of macromolecules such as proteins and trace cells. Compared with the data at 37 °C reported by Gabriel et al., there is a difference in the complex impedivity within 10 Hz–100 MHz. It is postulated that the discrepancy may arise from the fact that our study was conducted using human CSF, whereas Gabriel’s measurements were conducted using porcine CSF [10,11,12]. There is a significant difference in dielectric parameters between the species.

In order to provide an accurate description of change in CSF complex impedivity, it was first necessary to quantify the relationships among complex impedance, temperature, and frequency. A comparative analysis was conducted to evaluate the fitting efficacy of the two equivalent circuits depicted in Figure 1. The equivalent circuit depicted in Figure 1b was selected for further analysis as it exhibited a minimal fitting error and had more realistic characteristic parameters. On this basis, the change rule of each model parameter with temperature was analyzed. It was found that *R*_0_ and *R*_1_ decreased linearly with increasing temperature, and *f*_c_ increased linearly. *R*_0_ and *R*_1_’s temperature properties are related to the ionization degree of ions in the CSF, and *f*_c_’s temperature property may be related to the interactions between the current and the trace cells, proteins, and sugars. Once the functional relationship between each characteristic parameter and temperature has been determined, a final expression can be established between the CSF complex impedivity, temperature, and frequency based on the geometry of the tissue. The results of the fitting analysis indicated that the maximum discrepancy between the predictions of Equation (6) and the experimental data was 3.79%, which is an accurate representation of the changes in CSF dielectric properties with frequency and temperature.

Currently, CSF is examined for biochemical and cellular characteristics such as proteins, sugars, and cellular content to identify neurological disorders. However, these analyses are time-consuming and expensive [28]. The electrical impedance method is distinguished by its high sensitivity, rapid analysis, low cost, reliability, and the necessity of only small sample volumes. Moreover, it is very sensitive to alterations in the content of cells [29,30], proteins [31], and glucose [32] levels in biological tissues and has the potential to be applied to the analysis of CSF. Accordingly, this paper compared the differences in dielectric parameters between normal and pathological CSF at different temperatures and frequencies. The findings demonstrated that the absolute values of both the real and imaginary parts of pathological CSF were markedly higher than those observed in normal CSF. By comparing the biochemical indexes of the two types, it could be seen that the content of protein, white blood cells, erythrocyte and so on in pathological CSF increased significantly. The increase in these substances led to a deterioration in the conductive properties of CSF, thereby producing a significant difference in the dielectric parameters of the two types of CSF. Moreover, the analysis of the ROC curve for the real part at 10 Hz and 17 °C yielded an AUC of 0.898. The sensitivity was 0.944 and the specificity was 0.889 when identifying the CSF pathology. This finding preliminarily demonstrates the potential of the impedance method for identifying and pre-screening pathological CSF. The impedance method is expected to serve as a marker to understand the physical and chemical changes caused by brain lesions from more perspectives.

The temperature and frequency characteristics of the dielectric parameters of human CSF are rarely reported. Nevertheless, this study is of considerable importance in many fields such as the location and intensity evaluation of electrical generators within the brain, the modelling of electromagnetic fields within the human brain, specific absorption rate calculations, biological electromagnetic effects, and disease diagnosis [15,19,20,21]. Furthermore, this study is characterized by the following attributes: 1. Equation (6), describing the variation rule of CSF complex impedivity with temperature and frequency, is established for the first time. The formula adopts a concise mathematical expression and is capable of encompassing seven orders of magnitude between 10 Hz and 100 MHz, as well as a wide temperature range of 17–39 °C. Therefore, this enables researchers to perform precise calculations of the dielectric properties of CSF within this range. 2. The formula provides fundamental data for accurately integrating the relationship among the overall dielectric properties of the brain, temperature, and frequency. This will advance the research progress of EIT for the provision of early warnings of brain injury during CPB. 3. For the first time, we found that the dielectric parameters can be used to identify CSF lesions and quantified the accuracy of this identification. The alterations in the physical properties of CSF in the presence of lesions can be more deeply understood, providing new perspectives and research tools for disease diagnosis.

Although this study preliminarily explored the possibility of using dielectric parameters to distinguish between normal and pathological CSF with encouraging results, we recognize that this study has certain limitations. Firstly, due to the diverse types of pathological CSF and the variations in biochemical indicators, this study did not comprehensively investigate the potential of dielectric parameters in diagnosing various subtypes of CSF-related diseases. Moreover, the restricted number of pathological CSF samples precluded a comprehensive investigation into the relationship between dielectric parameters and key biochemical components (such as cell content, sugars, proteins, etc.) in CSF. To address these limitations, future studies will aim to collect more clinical samples and quantify the correlation between the dielectric parameters and biochemical components of CSF. This will facilitate the development of more precise models for identifying and classifying different types of pathological CSF. This will provide an important scientific foundation for the accurate diagnosis of central nervous system diseases and may facilitate the development of related diagnostic technologies.

## 5. Conclusions

The objective of this study was to investigate the frequency and temperature dependence of human CSF dielectric parameters at 10 Hz–100 MHz and 17–39 °C. Firstly, the comparison of dielectric parameters during cooling and reheating indicates that there is no statistically significant difference in the temperature dependence of CSF dielectric properties (*p* > 0.08). Based on this, an equation was developed to describe the relationships among CSF complex impedivity, temperature, and frequency, with a maximum fitting error of 3.79%. Finally, the dielectric parameters of CSF can be used to identify normal and pathological CSF. The ROC analysis yielded an AUC of 0.898, indicating a high degree of accuracy in this classification. The sensitivity was 0.944 and specificity was 0.889 when the real part of impedivity of 84.515 Ω·cm was used as the threshold for identifying CSF pathology. This study may facilitate the recognition of brain injury signals by EIT in the process of varying temperatures during CPB [7]. It may also provide a foundation for studies such as modelling and simulation studies of overall brain dielectric parameters’ temperature characteristics, biosensor design, non-invasive temperature monitoring, the dynamic monitoring of neurological injury treatment using EIT at variable temperatures [16], and the identification of CSF lesions using dielectric parameters.

## Figures and Tables

**Table 1:** Coefficients from the linear regression analysis of temperature-dependent parameters.

Characteristic Parameters	Slope (Ω/°C)	Constant Term (Ω)	Coefficient of Determination (*R*^2^)
$R_{0,fit}$ (Ω)	−9.836	795.172	0.990
$R_{1,fit}$ (Ω)	−9.758	770.002	0.990
$f_{c,fit}$ (Hz)	3,007,513.102	82,067,882.929	0.989

## Data Availability

The data presented in this study are available on request from the corresponding author due to privacy.

## References

[1] Hannan S., Faulkner M., Aristovich K., Avery J., Walker M.C., Holder D.S. (2020). In vivo imaging of deep neural activity from the cortical surface during hippocampal epileptiform events in the rat brain using electrical impedance tomography. Neuroimage.

[2] Abboud T., Hahn G., Just A., Paidhungat M., Nazarenus A., Mielke D., Rohde V. (2021). An insight into electrical resistivity of white matter and brain tumors. Brain Stimul..

[3] Hannan S., Faulkner M., Aristovich K., Avery J., Walker M., Holder D. (2018). Imaging fast electrical activity in the brain during ictal epileptiform discharges with electrical impedance tomography. Neuroimage-Clin..

[4] Ouypornkochagorn T., Polydorides N., McCann H. (2023). Towards continuous EIT monitoring for hemorrhagic stroke patients. Front. Physiol..

[5] Gaudino M., Benesch C., Bakaeen F., DeAnda A., Fremes S.E., Glance L., Messé S.R., Pandey A., Rong L.Q., Amer Heart Assoc Council C. (2020). Considerations for Reduction of Risk of Perioperative Stroke in Adult Patients Undergoing Cardiac and Thoracic Aortic Operations: A Scientific Statement From the American Heart Association. Circulation.

[6] Salameh A., Dhein S., Dähnert I., Klein N. (2016). Neuroprotective Strategies during Cardiac Surgery with Cardiopulmonary Bypass. Int. J. Mol. Sci..

[7] Li Y., Zhang D., Liu B., Jin Z., Duan W., Dong X., Fu F., Yu S., Shi X. (2018). Noninvasive Cerebral Imaging and Monitoring Using Electrical Impedance Tomography During Total Aortic Arch Replacement. J. Cardiothor. Vasc. Anesth..

[8] Latikka J., Kuurne T., Eskola H. (2001). Conductivity of living intracranial tissues. Phys. Med. Biol..

[9] Koessler L., Colnat-Coulbois S., Cecchin T., Hofmanis J., Dmochowski J.P., Norcia A.M., Maillard L.G. (2017). In-vivo measurements of human brain tissue conductivity using focal electrical current injection through intracerebral multicontact electrodes. Hum. Brain Mapp..

[10] Gabriel S., Lau R.W., Gabriel C. (1996). The dielectric properties of biological tissues: III. Parametric models for the dielectric spectrum of tissues. Phys. Med. Biol..

[11] Gabriel C., Peyman A., Grant E.H. (2009). Electrical conductivity of tissue at frequencies below 1 MHz. Phys. Med. Biol..

[12] Peyman A., Holden S.J., Watts S., Perrott R., Gabriel C. (2007). Dielectric properties of porcine cerebrospinal tissues at microwave frequencies:in vivo, in vitro and systematic variation with age. Phys. Med. Biol..

[13] Gabriel C., Gabriel S., Corthout E. (1996). The dielectric properties of biological tissues: I. Literature survey. Phys. Med. Biol..

[14] Gabriel S., Lau R.W., Gabriel C. (1996). The dielectric properties of biological tissues: II. Measurements in the frequency range 10 Hz to 20 GHz. Phys. Med. Biol..

[15] Baumann S.B., Wozny D.R., Kelly S.K., Meno F.M. (1997). The electrical conductivity of human cerebrospinal fluid at body temperature. IEEE Trans. Biomed. Eng..

[16] Lingwood B.E., Dunster K.R., Healy G.N., Colditz P.B. (2004). Effect of cooling and re-warming on cerebral and whole body electrical impedance. Physiol. Meas..

[17] Thrane A.S., Rangroo Thrane V., Nedergaard M. (2014). Drowning stars: Reassessing the role of astrocytes in brain edema. Trends Neurosci..

[18] Gibson A., Bayford R.H., Holder D.S. (2000). Two-dimensional finite element modelling of the neonatal head. Physiol. Meas..

[19] Huang J.C., Nicholson C., Okada Y.C. (1990). Distortion of magnetic evoked fields and surface potentials by conductivity differences at boundaries in brain tissue. Biophys. J..

[20] Lascano A.M., Vulliemoz S., Lantz G., Spinelli L., Michel C., Seeck M. (2012). A review on non-invasive localisation of focal epileptic activity using EEG source imaging. Epileptologie.

[21] Yi G.S., Wang J., Wei X., Deng B., Tsang K.M., Chan W.L., Han C.X. (2014). Effects of extremely low-frequency magnetic fields on the response of a conductance-based neuron model. Int. J. Neural Syst..

[22] Wang W., Li W., Liu B., Wang L., Li K., Wang Y., Ji Z., Xu C., Shi X. (2022). Temperature dependence of dielectric properties of blood at 10 Hz–100 MHz. Front. Physiol..

[23] Truong B.C., Tuan H.D., Fitzgerald A.J., Wallace V.P., Nguyen H.T. (2015). A dielectric model of human breast tissue in terahertz regime. IEEE Trans. Biomed. Eng..

[24] Yang B., Xu J., Hu S., You B., Ma Q. (2021). Effects of lead exposure on blood electrical impedance spectroscopy of mice. Biomed. Eng. Online.

[25] Gregory W.D., Marx J.J., Gregory C.W., Mikkelson W.M., Tjoe J.A., Shell J. (2012). The Cole relaxation frequency as a parameter to identify cancer in breast tissue. Med. Phys..

[26] Tsai J.Y., Pan W., LeMaire S.A., Pisklak P., Lee V.V., Bracey A.W., Elayda M.A., Preventza O., Price M.D., Collard C.D. (2013). Moderate hypothermia during aortic arch surgery is associated with reduced risk of early mortality. J. Thorac. Cardiovasc. Sur..

[27] Zhbanov A., Yang S. (2020). Electrochemical Impedance Characterization of Blood Cell Suspensions—Part 2: Three-Phase Systems With Single-Shelled Particles. IEEE Trans. Biomed. Eng..

[28] Rajasekharan C., Girishkumar C., Lonappan A., Mathew A.J., Mathew K.T. (2010). Diagnostic Value of Microwaves in Neurological Disorders. J. Microw. Power Electromagn. Energy.

[29] Zhu C.Z., Ting H.N., Ng K.H., Mun K.S., Ong T.A. (2023). Dielectric properties of urine in relation to bladder cancer. Phys. Eng. Sci. Med..

[30] Kadan-Jamal K., Sophocleous M., Jog A., Desagani D., Teig-Sussholz O., Georgiou J., Avni A., Shacham-Diamand Y. (2020). Electrical Impedance Spectroscopy of plant cells in aqueous biological buffer solutions and their modelling using a unified electrical equivalent circuit over a wide frequency range: 4Hz to 20 GHz. Biosens. Bioelectron..

[31] Basey-Fisher T.H., Guerra N., Triulzi C., Gregory A., Hanham S.M., Stevens M.M., Maier S.A., Klein N. (2014). Microwaving blood as a non-destructive technique for haemoglobin measurements on microlitre samples. Adv. Healthc. Mater..

[32] Pedro B.G., Marcôndes D.W.C., Bertemes-Filho P. (2020). Analytical Model for Blood Glucose Detection Using Electrical Impedance Spectroscopy. Sensors.

